# Statistical analysis of differential equations: introducing probability measures on numerical solutions

**DOI:** 10.1007/s11222-016-9671-0

**Published:** 2016-06-02

**Authors:** Patrick R. Conrad, Mark Girolami, Simo Särkkä, Andrew Stuart, Konstantinos Zygalakis

**Affiliations:** 1 Department of Statistics, University of Warwick, Coventry, UK; 2 Present Address: Alan Turing Institute, London, UK; 3 Department of Electrical Engineering and Automation, Aalto University, Espoo, Finland; 4 Department of Mathematics, University of Warwick, Coventry, UK; 5 School of Mathematics, University of Edinburgh, Edinburgh, Scotland

**Keywords:** Numerical analysis, Probabilistic numerics, Inverse problems, Uncertainty quantification, 62F15, 65N75, 65L20

## Abstract

In this paper, we present a formal quantification of uncertainty induced by numerical solutions of ordinary and partial differential equation models. Numerical solutions of differential equations contain inherent uncertainties due to the finite-dimensional approximation of an unknown and implicitly defined function. When statistically analysing models based on differential equations describing physical, or other naturally occurring, phenomena, it can be important to explicitly account for the uncertainty introduced by the numerical method. Doing so enables objective determination of this source of uncertainty, relative to other uncertainties, such as those caused by data contaminated with noise or model error induced by missing physical or inadequate descriptors. As ever larger scale mathematical models are being used in the sciences, often sacrificing complete resolution of the differential equation on the grids used, formally accounting for the uncertainty in the numerical method is becoming increasingly more important. This paper provides the formal means to incorporate this uncertainty in a statistical model and its subsequent analysis. We show that a wide variety of existing solvers can be randomised, inducing a probability measure over the solutions of such differential equations. These measures exhibit contraction to a Dirac measure around the true unknown solution, where the rates of convergence are consistent with the underlying deterministic numerical method. Furthermore, we employ the method of modified equations to demonstrate enhanced rates of convergence to stochastic perturbations of the original deterministic problem. Ordinary differential equations and elliptic partial differential equations are used to illustrate the approach to quantify uncertainty in both the statistical analysis of the forward and inverse problems.

**Electronic supplementary material:**

The online version of this article (doi:10.1007/s11222-016-9671-0) contains supplementary material, which is available to authorized users.

## Introduction

### Motivation

The numerical analysis literature has developed a large range of efficient algorithms for solving ordinary and partial differential equations, which are typically designed to solve a single problem as efficiently as possible (Hairer et al. 1993; Eriksson 1996). When classical numerical methods are placed within statistical analysis, however, we argue that significant difficulties can arise as a result of errors in the computed approximate solutions. While the distributions of interest commonly do converge asymptotically as the solver mesh becomes dense [e.g. in statistical inverse problems (Dashti and Stuart 2016)], we argue that at a finite resolution, the statistical analyses may be vastly overconfident as a result of these unmodelled errors.

The purpose of this paper is to address these issues by the construction and rigorous analysis of novel probabilistic integration methods for both ordinary and partial differential equations. The approach in both cases is similar: we identify the key discretisation assumptions and introduce a local random field, in particular a Gaussian field, to reflect our uncertainty in those assumptions. The probabilistic solver may then be sampled repeatedly to interrogate the uncertainty in the solution. For a wide variety of commonly used numerical methods, our construction is straightforward to apply and provably preserves the order of convergence of the original method.

Furthermore, we demonstrate the value of these probabilistic solvers in statistical inference settings. Analytic and numerical examples show that using a classical non-probabilistic solver with inadequate discretisation when performing inference can lead to inappropriate and misleading posterior concentration in a Bayesian setting. In contrast, the probabilistic solver reveals the structure of uncertainty in the solution, naturally limiting posterior concentration as appropriate.

**Fig. 1:**
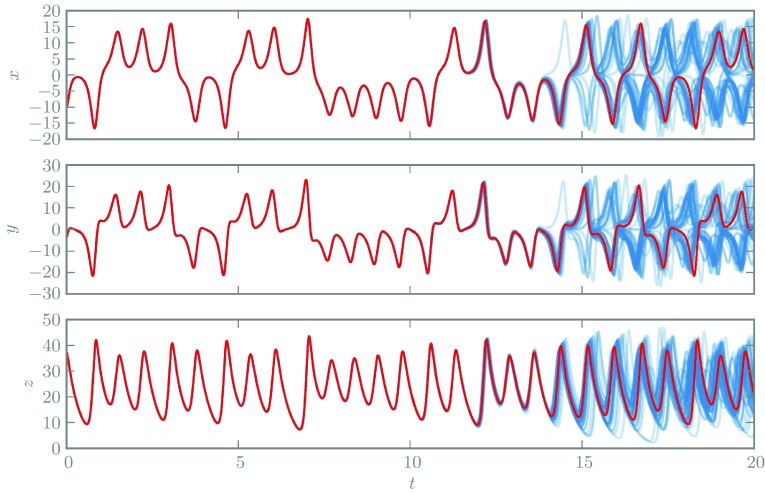
A comparison of solutions to the Lorenz’63 system using deterministic (*red*) and randomised (*blue*) integrators based on a fourth-order Runge–Kutta integrator

As a motivating example, consider the solution of the Lorenz’63 system. Since the problem is chaotic, any typical fixed-step numerical methods will become increasingly inaccurate for long integration times. Figure 1 depicts a deterministic solution for this problem, computed with a fixed-step, fourth-order, Runge–Kutta integrator. Although the solver becomes completely inaccurate by the end of the depicted interval given the step-size selected, the solver provides no obvious characterisation of its error at late times. Compare this with a sample of randomised solutions based on the same integrator and the same step-size; it is obvious that early-time solutions are accurate and that they diverge at late times, reflecting instability of the solver. Every curve drawn has the same theoretical accuracy as the original classical method, but the randomised integrator provides a detailed and practical approach for revealing the sensitivity of the solution to numerical errors. The method used requires only a straightforward modification of the standard Runge–Kutta integrator and is explained in Sect. 2.3.

We summarise the contributions of this work as follows:

- Construct randomised solvers of ODEs and PDEs using natural modification of popular, existing solvers.
- Prove the convergence of the randomised methods and study their behaviour by showing a close link between randomised ODE solvers and stochastic differential equations (SDEs).
- Demonstrate that these randomised solvers can be used to perform statistical analyses that appropriately consider solver uncertainty.

### Review of existing work

The statistical analysis of models based on ordinary and partial differential equations is growing in importance and a number of recent papers in the statistics literature have sought to address certain aspects specific to such models, e.g. parameter estimation (Liang and Wu 2008; Xue et al. 2010; Xun et al. 2013; Brunel et al. 2014) and surrogate construction (Chakraborty et al. 2013). However, the statistical implications of the reliance on a numerical approximation to the actual solution of the differential equation have not been addressed in the statistics literature to date and this is the open problem comprehensively addressed in this paper. Earlier work in the literature including randomisation in the approximate integration of ordinary differential equations (ODEs) includes (Coulibaly and Lécot 1999; Stengle 1995). Our strategy fits within the emerging field known as Probabilistic Numerics (Hennig et al. 2015), a perspective on computational methods pioneered by Diaconis (1988), and subsequently (Skilling 1992). This framework recasts solving differential equations as a statistical inference problem, yielding a probability measure over functions that satisfy the constraints imposed by the specific differential equation. This measure formally quantifies the uncertainty in candidate solution(s) of the differential equation, allowing its use in uncertainty quantification (Sullivan 2016) or Bayesian inverse problems (Dashti and Stuart 2016).

A recent Probabilistic Numerics methodology for ODEs (Chkrebtii et al. 2013) [explored in parallel in Hennig and Hauberg (2014)] has two important shortcomings. First, it is impractical, only supporting first-order accurate schemes with a rapidly growing computational cost caused by the growing difference stencil [although Schober et al. (2014) extends to Runge–Kutta methods]. Secondly, this method does not clearly articulate the relationship between their probabilistic structure and the problem being solved. These methods construct a Gaussian process whose mean coincides with an existing deterministic integrator. While they claim that the posterior variance is useful, by the conjugacy inherent in linear Gaussian models, it is actually just an *a priori* estimate of the rate of convergence of the integrator, independent of the actual forcing or initial condition of the problem being solved. These works also describe a procedure for randomising the construction of the mean process, which bears similarity to our approach, but it is not formally studied. In contrast, we formally link each draw from our measure to the analytic solution.

Our motivation for enhancing inference problems with models of discretisation error is similar to the more general concept of model error, as developed by Kennedy and O’Hagan (2001). Although more general types of model error, including uncertainty in the underlying physics, are important in many applications, our focus on errors arising from the discretisation of differential equations leads to more specialised methods. Future work may be able to translate insights from our study of the restricted problem to the more general case. Existing strategies for discretisation error include empirically fitted Gaussian models for PDE errors (Kaipio and Somersalo 2007) and randomly perturbed ODEs (Arnold et al. 2013); the latter partially coincides with our construction, but our motivation and analysis are distinct. Recent work (Capistrán et al. 2013) uses Bayes factors to analyse the impact of discretisation error on posterior approximation quality. Probabilistic models have also been used to study error propagation due to rounding error; see Hairer et al. (2008).

### Organisation

The remainder of the paper has the following structure: Sect. 2 introduces and formally analyses the proposed probabilistic solvers for ODEs. Section 3 explores the characteristics of random solvers employed in the statistical analysis of both forward and inverse problems. Then, we turn to elliptic PDEs in Sect. 4, where several key steps of the construction of probabilistic solvers and their analysis have intuitive analogues in the ODE context. Finally, an illustrative example of an elliptic PDE inference problem is presented in Sect. 5.^1^

## Probability measures via probabilistic time integrators

Consider the following ordinary differential equation (ODE):

1 $$\begin{aligned} \frac{\mathrm{d}u}{\mathrm{d}t}=f(u), \quad u(0)=u_{0}, \end{aligned}$$

where $u(\cdot )$ is a continuous function taking values in $\mathbb {R}^n$.^2^ We let $\varPhi _t$ denote the flow map for Eq. (1), so that $u(t)=\varPhi _t\bigl (u(0)\bigr )$. The conditions ensuring that this solution exists will be formalised in Assumption 2, below.

Deterministic numerical methods for the integration of this equation on time interval [0, *T*] will produce an approximation to the equation on a mesh of points $\{t_k=kh\}_{k=0}^{K}$, with $Kh=T$, (for simplicity we assume a fixed mesh). Let $u_k=u(t_k)$ denote the exact solution of (1) on the mesh and $U_k\approx u_k$ denote the approximation computed using finite evaluations of *f*. Typically, these methods output a single discrete solution $\{U_k\}_{k=0}^K$, often augmented with some type of error indicator, but do not statistically quantify the uncertainty remaining in the path.

Let $X_{a,b}$ denote the Banach space $C([a,b];\mathbb {R}^n)$. The exact solution of (1) on the time interval [0, *T*] may be viewed as a Dirac measure $\delta _u$ on $X_{0,T}$ at the element *u* that solves the ODE. We will construct a probability measure $\mu ^h$ on $X_{0,T}$, that is straightforward to sample from both on and off the mesh, for which *h* quantifies the size of the discretisation step employed, and whose distribution reflects the uncertainty resulting from the solution of the ODE. Convergence of the numerical method is then related to the contraction of $\mu ^h$ to $\delta _u$.

We briefly summarise the construction of the numerical method. Let $\varPsi _{h}:\mathbb {R}^n \rightarrow \mathbb {R}^n$ denote a classical deterministic one-step numerical integrator over time-step *h*, a class including all Runge–Kutta methods and Taylor methods for ODE numerical integration (Hairer et al. 1993). Our numerical methods will have the property that, on the mesh, they take the form

2 $$\begin{aligned} U_{k+1}=\varPsi _{h}(U_k)+\xi _k(h), \end{aligned}$$

where $\xi _k(h)$ are suitably scaled, i.i.d. Gaussian random variables. That is, the random solution iteratively takes the standard step, $\varPsi _{h}$, followed by perturbation with a random draw, $\xi _k(h)$, modelling uncertainty that accumulates between mesh points. The discrete path $\{U_k\}_{k=0}^K$ is straightforward to sample and in general is not a Gaussian process. Furthermore, the discrete trajectory can be extended into a continuous time approximation of the ODE, which we define as a draw from the measure $\mu ^h$.

The remainder of this section develops these solvers in detail and proves strong convergence of the random solutions to the exact solution, implying that $\mu ^h \rightarrow \delta _u$ in an appropriate sense. Finally, we establish a close relationship between our random solver and a stochastic differential equation (SDE) with small mesh-dependent noise. Intuitively, adding Gaussian noise to an ODE suggests a link to SDEs. Additionally, note that the mesh-restricted version of our algorithm, given by (2), has the same structure as a first-order Ito–Taylor expansion of the SDE

3 $$\begin{aligned} \mathrm{d}u = f(u) \mathrm{d}t + \sigma \mathrm{d}W, \end{aligned}$$

for some choice of $\sigma$. We make this link precise by performing a backwards error analysis, which connects the behaviour of our solver to an associated SDE.

### Probabilistic time integrators: general formulation

The integral form of Eq. (1) is

4 $$\begin{aligned} u(t)=u_0+\int _{0}^{t}f\bigl (u(s)\bigr )\mathrm{d}s. \end{aligned}$$

The solutions on the mesh satisfy

5 $$\begin{aligned} u_{k+1}=u_k+\int _{t_k}^{t_{k+1}}f\bigl (u(s)\bigr )\mathrm{d}s, \end{aligned}$$

and may be interpolated between mesh points by means of the expression

6 $$\begin{aligned} u(t)=u_k+\int _{t_k}^{t}f\bigl (u(s)\bigr )\mathrm{d}s, \quad t \in [t_k,t_{k+1}). \end{aligned}$$

We may then write

7 $$\begin{aligned} u(t)=u_{k}+\int _{t_k}^{t}g(s)\mathrm{d}s, \quad t \in [t_k,t_{k+1}), \end{aligned}$$

where $g(s)=f\bigl (u(s)\bigr )$ is an unknown function of time. In the algorithmic setting, we have approximate knowledge about *g*(*s*) through an underlying numerical method. A variety of traditional numerical algorithms may be derived based on approximation of *g*(*s*) by various simple deterministic functions $g^h(s)$. The simplest such numerical method arises from invoking the Euler approximation that

8 $$\begin{aligned} g^h(s)=f(U_{k}), \quad s \in [t_k,t_{k+1}). \end{aligned}$$

In particular, if we take $t=t_{k+1}$ and apply this method inductively the corresponding numerical scheme arising from making such an approximation to *g*(*s*) in (7) is $U_{k+1}=U_{k}+hf(U_{k}).$ Now consider the more general one-step numerical method $U_{k+1}=\varPsi _{h}(U_k).$ This may be derived by approximating *g*(*s*) in (7) by9We note that all consistent (in the sense of numerical analysis) one-step methods will satisfy

$$\begin{aligned} \frac{\mathrm{d}}{\mathrm{d}\tau }\Bigl (\varPsi _{\tau }(u)\Bigr )_{\tau =0}=f(u). \end{aligned}$$

The approach based on the approximation (9) leads to a deterministic numerical method which is defined as a continuous function of time. Specifically, we have $U(s)=\varPsi _{s-t_k}(U_k), \quad s \in [t_k,t_{k+1}).$ Consider again the Euler approximation, for which $\varPsi _{\tau }(U)=U+\tau f(U)$, and whose continuous time interpolant is then given by $U(s)=U_k+(s-t_k)f(U_k), \;\; s \in [t_k,t_{k+1}).$ Note that this produces a continuous function, namely an element of $X_{0,T}$, when extended to $s \in [0,T].$ The preceding development of a numerical integrator does not acknowledge the uncertainty that arises from lack of knowledge about *g*(*s*) in the interval $s \in [t_k,t_{k+1}).$ We propose to approximate *g* stochastically in order to represent this uncertainty, taking

$$\begin{aligned} g^h(s)= \frac{\mathrm{d}}{\mathrm{d}\tau }\Bigl (\varPsi _{\tau }(U_k)\Bigr )_{\tau =s-t_k}+\chi _k(s-t_k), \quad s \in [t_k,t_{k+1}) \end{aligned}$$

where the $\{\chi _k\}$ form an i.i.d. sequence of Gaussian random functions defined on [0, *h*] with $\chi _k \sim N(0,C^h)$.^3^

We will choose $C^h$ to shrink to zero with *h* at a prescribed rate (see Assumption 1), and also to ensure that $\chi _k \in X_{0,h}$ almost surely. The functions $\{\chi _k\}$ represent our uncertainty about the function *g*. The corresponding numerical scheme arising from such an approximation is given by

10 $$\begin{aligned} U_{k+1}=\varPsi _{h}(U_{k})+\xi _k(h), \end{aligned}$$

where the i.i.d. sequence of functions $\{\xi _k\}$ lies in $X_{0,h}$ and is given by

11 $$\begin{aligned} \xi _k(t)=\int _{0}^{t}\chi _{k}(\tau )\mathrm{d}\tau . \end{aligned}$$

Note that the numerical solution is now naturally defined between grid points, via the expression

12 $$\begin{aligned} U(s)=\varPsi _{s-t_k}(U_{k})+\xi _k(s-t_k), \quad s \in [t_k,t_{k+1}). \end{aligned}$$

When it is necessary to evaluate a solution at multiple points in an interval, $s \in (t_k,t_{k+1}]$, the perturbations $\xi _k(s-t_k)$ must be drawn jointly, which is facilitated by their Gaussian structure. Although most users will only need the formulation on mesh points, we must consider off-mesh behaviour to rigorously analyse higher order methods, as is also required for the deterministic variants of these methods.

In the case of the Euler method, for example, we have

13 $$\begin{aligned} U_{k+1}=U_{k}+hf(U_{k})+\xi _k(h) \end{aligned}$$

and, between grid points,

14 $$\begin{aligned} U(s)=U_{k}\,+\,(s\,-\,t_k)f(U_{k})\,+\,\xi _k(s-t_k), \quad s \in [t_k,t_{k+1}). \end{aligned}$$

**Fig. 2:**
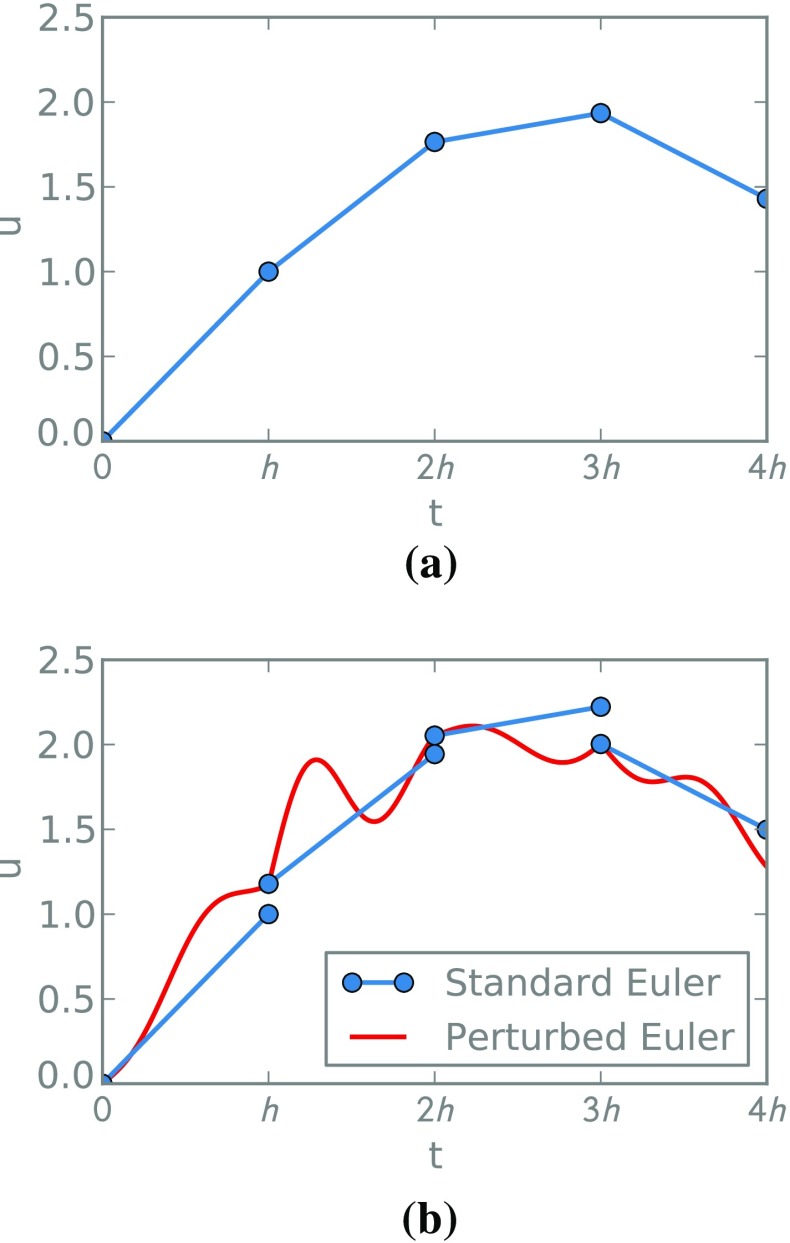
An illustration of deterministic Euler steps and randomised variations. The random integrator in (**b**) outputs the path in red; we overlay the standard Euler step constructed at each step, before it is perturbed (*blue*)

This method is illustrated in Fig. 2. Observe that Eq. (13) has the same form as an Euler–Maryama method for an associated SDE (3) where $\sigma$ depends on the step-size *h*. In particular, in the simple one-dimensional case, $\sigma$ would be given by $\sqrt{C^{h}/h}$. Section 2.4 develops a more sophisticated connection that extends to higher order methods and off the mesh.

While we argue that the choice of modelling local uncertainty in the flow map as a Gaussian process is natural and analytically favourable, it is not unique. It is possible to construct examples where the Gaussian assumption is invalid; for example, when a highly inadequate time-step is used, a systemic bias may be introduced. However, in regimes where the underlying deterministic method performs well, the centred Gaussian assumption is a reasonable prior.

### Strong convergence result

To prove the strong convergence of our probabilistic numerical solver, we first need two assumptions quantifying properties of the random noise and of the underlying deterministic integrator, respectively. In what follows we use $\langle \cdot , \cdot \rangle$ and $|\cdot |$ to denote the Euclidean inner product and norm on $\mathbb {R}^n$. We denote the Frobenius norm on $\mathbb {R}^{n \times n}$ by $|\cdot |_\mathrm{F}$, and $\mathbb {E}^h$ denotes expectation with respect to the i.i.d. sequence $\{\chi _k\}$.

#### Assumption 1

Let $\xi _k(t)\!:=\int _0^t \chi _k(s)ds$ with $\chi _k \sim N(0,C^h).$ Then there exists $K>0, p \ge 1$ such that, for all $t \in [0,h]$, $\mathbb {E}^h|\xi _k(t) \xi _k(t)^T|_\mathrm{F}^2 \le Kt^{2p+1};$ in particular $\mathbb {E}^h|\xi _k(t)|^2 \le Kt^{2p+1}.$ Furthermore, we assume the existence of matrix *Q*, independent of *h*, such that $\mathbb {E}^h[\xi _k(h) \xi _k(h)^T]=Qh^{2p+1}.$

Here, and in the sequel, *K* is a constant independent of *h*, but possibly changing from line to line. Note that the covariance kernel $C^h$ is constrained, but not uniquely defined. We will assume the form of the constant matrix is $Q=\sigma I$, and we discuss one possible strategy for choosing $\sigma$ in Sect. 3.1. Section 2.4 uses a weak convergence analysis to argue that once *Q* is selected, the exact choice of $C^h$ has little practical impact.

#### Assumption 2

The function *f* and a sufficient number of its derivatives are bounded uniformly in $\mathbb {R}^n$ in order to ensure that *f* is globally Lipschitz and that the numerical flow map $\varPsi _h$ has uniform local truncation error of order $q+1$:

$$\begin{aligned} \sup _{u \in \mathbb {R}^n} |\varPsi _t(u)-\varPhi _t(u)| \le Kt^{q+1}. \end{aligned}$$

#### Remark 2.1

We assume globally Lipschitz *f*, and bounded derivatives, in order to highlight the key probabilistic ideas, whilst simplifying the numerical analysis. Future work will address the non-trivial issue of extending of analyses to weaken these assumptions. In this paper, we provide numerical results indicating that a weakening of the assumptions is indeed possible.

#### Theorem 2.2

Under Assumptions 1, 2 it follows that there is $K>0$ such that

$$\begin{aligned} \sup _{0 \le kh \le T}\mathbb {E}^h|u_k-U_k|^2 \le Kh^{2\min \{p,q\}}. \end{aligned}$$

Furthermore,

$$\begin{aligned} \sup _{0 \le t \le T}\mathbb {E}^h|u(t)-U(t)| \le Kh^{\min \{p,q\}}. \end{aligned}$$

This theorem implies that every probabilistic solution is a good approximation of the exact solution in both a discrete and continuous sense. Choosing $p \ge q$ is natural if we want to preserve the strong order of accuracy of the underlying deterministic integrator; we proceed with the choice $p=q$, introducing the maximum amount of noise consistent with this constraint.

### Examples of probabilistic time integrators

The canonical illustration of a probabilistic time integrator is the probabilistic Euler method already described.^4^ Another useful example is the classical *Runge–Kutta method* which defines a one-step numerical integrator as follows:

$$\begin{aligned} \varPsi _{h}(u)=u+\frac{h}{6}\bigl (k_1(u)+2k_2(u,h)+2k_3(u,h)+k_4(u,h)\bigr ), \end{aligned}$$

where

$$\begin{aligned}&k_1(u)=f(u),\quad k_2(u,h)=f\bigl (u+\frac{1}{2}h k_1(u)\bigr )\\&k_3(u,h)=f\bigl (u+\frac{1}{2}h k_2(u)\bigr ),\quad k_4(u,h)=f\bigl (u+h k_3(u)\bigr ). \end{aligned}$$

The method has local truncation error in the form of Assumption 2 with $q=4.$ It may be used as the basis of a probabilistic numerical method (12), and hence (10) at the grid points. Thus, provided that we choose to perturb this integrator with a random process $\chi _k$ satisfying Assumption 1 with $p \ge 4$,^5^ Theorem 2.2 shows that the error between the probabilistic integrator based on the classical Runge–Kutta method is, in the mean square sense, of the same order of accuracy as the deterministic classical Runge–Kutta integrator.

### Backward error analysis

Backwards error analyses are useful tool for numerical analysis; the idea is to characterise the method by identifying a modified equation (dependent upon *h*) which is solved by the numerical method either exactly, or at least to a higher degree of accuracy than the numerical method solves the original equation. For our random ODE solvers, we will show that the modified equation is a stochastic differential equation (SDE) in which only the matrix *Q* from Assumption 1 enters; the details of the random processes used in our construction do not enter the modified equation. This universality property underpins the methodology we introduce as it shows that many different choices of random processes all lead to the same effective behaviour of the numerical method.

We introduce the operators $\mathcal {L}$ and $\mathcal {L}^h$ defined so that, for all $\phi \in C^{\infty }(\mathbb {R}^n,\mathbb {R})$,

15 $$\begin{aligned} \phi \bigl (\varPhi _h(u)\bigr )=\bigl (e^{h\mathcal {L}}\phi \bigr )(u), \quad \mathbb {E}\phi \bigl (U_1|U_0=u\bigr )=\bigl (e^{h\mathcal {L}^h}\phi \bigr )(u). \end{aligned}$$

Thus $\mathcal {L}:=f \cdot \nabla$ and $e^{h\mathcal {L}^h}$ is the kernel for the Markov chain generated by the probabilistic integrator (2). In fact we never need to work with $\mathcal {L}^h$ itself in what follows, only with $e^{h\mathcal {L}^h}$, so that questions involving the operator logarithm do not need to be discussed.

We now introduce a modified ODE and a modified SDE which will be needed in the analysis that follows. The modified ODE is

16 $$\begin{aligned} \frac{\mathrm{d}\hat{u}}{\mathrm{d}t}=f^h(\hat{u}) \end{aligned}$$

whilst the modified SDE has the form

17 $$\begin{aligned} \mathrm{d}\tilde{u}=f^h(\tilde{u})\mathrm{d}t+\sqrt{h^{2p} Q} \, \mathrm{d}W. \end{aligned}$$

The precise choice of $f^h$ is detailed below. Letting $\mathbb {E}$ denote expectation with respect to *W*, we introduce the operators $\widehat{\mathcal {L}}^h$ and $\widetilde{\mathcal {L}}^h$ so that, for all $\phi \in C^{\infty }(\mathbb {R}^n,\mathbb {R})$,

18 $$\begin{aligned}&\phi \bigl (\hat{u}(h)|\hat{u}(0)=u\bigr )=\bigl (e^{h\widehat{\mathcal {L}}^h}\phi \bigr )(u), \end{aligned}$$

19 $$\begin{aligned}&\mathbb {E}\phi \bigl (\tilde{u}(h)|\tilde{u}(0)=0\bigr )=\bigl (e^{h\widetilde{\mathcal {L}}^h}\phi \bigr )(u). \end{aligned}$$

Thus,

20 $$\begin{aligned} \widehat{\mathcal {L}}^h:=f^h \cdot \nabla , \quad \widetilde{\mathcal {L}}^h=f^h \cdot \nabla +\frac{1}{2}h^{2p}Q:\nabla \nabla , \end{aligned}$$

where  :  denotes the inner product on $\mathbb {R}^{n \times n}$ which induces the Frobenius norm, that is, *A*:*B* $= \text {trace}(A^{T} B)$.

The fact that the deterministic numerical integrator has uniform local truncation error of order $q+1$ (Assumption 2) implies that, since $\phi \in C^{\infty }$,

21 $$\begin{aligned} e^{h \mathcal {L}}\phi (u)-\phi (\varPsi _{h}(u))=\mathcal {O}(h^{q+1}). \end{aligned}$$

The theory of modified equations for classical one-step numerical integration schemes for ODEs (Hairer et al. 1993) establishes that it is possible to find $f^h$ in the form

22 $$\begin{aligned} f^h:=f+\sum _{i=q}^{q+l}h^{i}f_{i}, \end{aligned}$$

such that

23 $$\begin{aligned} e^{h \widehat{\mathcal {L}}^h}\phi (u)-\phi (\varPsi _{h}(u))=\mathcal {O}(h^{q+2+l}). \end{aligned}$$

We work with this choice of $f^h$ in what follows.

Now for our stochastic numerical method we have

$$\begin{aligned} \phi (U_{k+1})= & {} \phi (\varPsi _{h}(U_{k}))+\xi _{k}(h) \cdot \nabla \phi (\varPsi _{h}(U_{k}))\\&+\,\frac{1}{2} \xi _{k}(h)\xi ^{T}_{k}(h) : \nabla \nabla \phi (\varPsi _{h}(U_{k})) + {\mathcal {O}}(|\xi _k(h)|^3). \end{aligned}$$

Furthermore, the last term has mean of size ${\mathcal {O}}(|\xi _k(h)|^4)$. From Assumption 1 we know that $\mathbb {E}^h\left( \xi _{k}(h)\xi ^{T}_{k}(h) \right) = Q h^{2p+1}.$ Thus

24 $$\begin{aligned}&e^{h\mathcal {L}^h}\phi (u) - \phi \bigl (\varPsi _{h}(u)\bigr )\nonumber \\&\quad = \frac{1}{2} h^{2p+1} Q : \nabla \nabla \phi \bigl (\varPsi _{h}(u)\bigr )+\mathcal {O}(h^{4p+2}). \end{aligned}$$

From this it follows that

25 $$\begin{aligned}&e^{h\mathcal {L}^h}\phi (u) - \phi \bigl (\varPsi _{h}(u)\bigr )\nonumber \\&\quad = \frac{1}{2} h^{2p+1} Q : \nabla \nabla \phi (u)+\mathcal {O}(h^{2p+2}). \end{aligned}$$

Finally we note that (20) implies that

$$\begin{aligned}&e^{h\widetilde{\mathcal {L}}^h}\phi (u)-e^{h\widehat{\mathcal {L}}^h}\phi (u)\\&\quad = e^{h\widehat{\mathcal {L}}^h}\bigl (e^{\frac{1}{2} h^{2p+1}Q:\nabla \nabla }-I\bigr )\phi (u)\\&\quad =e^{h\widehat{\mathcal {L}}^h}\Bigl (\frac{1}{2} h^{2p+1}Q:\nabla \nabla \phi (u)+\mathcal {O}(h^{4p+2})\Bigr )\\&\quad =\bigl (I+\mathcal {O}(h)\bigr )\Bigl (\frac{1}{2} h^{2p+1}Q:\nabla \nabla \phi (u)\\&\qquad +\mathcal {O}(h^{4p+2})\Bigr ). \end{aligned}$$

Thus we have

26 $$\begin{aligned} e^{h\widetilde{\mathcal {L}}^h}\phi (u)-e^{h\widehat{\mathcal {L}}^h}\phi (u)=\frac{1}{2} h^{2p+1}Q:\nabla \nabla \phi (u)+\mathcal {O}(h^{2p+2}). \end{aligned}$$

Now using (23), (25), and (26) we obtain

27 $$\begin{aligned} e^{h\widetilde{\mathcal {L}}^h}\phi (u)-e^{h\mathcal {L}^h}\phi (u)=\mathcal {O}(h^{2p+2})+\mathcal {O}(h^{q+2+l}). \end{aligned}$$

Balancing these terms, in what follows we make the choice $l=2p-q$. If $l<0$ we adopt the convention that the drift $f^h$ is simply *f*. With this choice of *q* we obtain

28 $$\begin{aligned} e^{h\widetilde{\mathcal {L}}^h}\phi (u)-e^{h\mathcal {L}^h}\phi (u)=\mathcal {O}(h^{2p+2}). \end{aligned}$$

This demonstrates that the error between the Markov kernel of one-step of the SDE (17) and the Markov kernel of the numerical method (2) is of order $\mathcal {O}(h^{2p+2})$. Some straightforward stability considerations show that the weak error over an $\mathcal {O}(1)$ time interval is $\mathcal {O}(h^{2p+1})$. We make assumptions giving this stability and then state a theorem comparing the weak error with respect to the modified Eq. (17), and the original Eq. (1).

#### Assumption 3

The function *f* is in $C^{\infty }$ and all its derivatives are uniformly bounded on $\mathbb {R}^n$. Furthermore, *f* is such that the operators $e^{h\mathcal {L}}$ and $e^{h\mathcal {L}^h}$ satisfy, for all $\psi \in C^{\infty }(\mathbb {R}^n,\mathbb {R})$ and some $L>0$,

$$\begin{aligned} \sup _{u \in \mathbb {R}^n}|e^{h\mathcal {L}}\psi (u)|&\le (1+Lh)\sup _{u \in \mathbb {R}^n}|\psi (u)|,\\ \sup _{u \in \mathbb {R}^n}|e^{h\mathcal {L}^h}\psi (u)|&\le (1+Lh)\sup _{u \in \mathbb {R}^n}|\psi (u)|. \end{aligned}$$

#### Remark 2.3

If $p=q$ in what follows (our recommended choice) then the weak order of the method coincides with the strong order; however, measured relative to the modified equation, the weak order is then one plus twice the strong order. In this case, the second part of Theorem 2.2 gives us the first weak order result in Theorem 2.4. Additionally, Assumption 3 is stronger than we need, but allows us to highlight probabilistic ideas whilst keeping overly technical aspects of the numerical analysis to a minimum. More sophisticated, but structurally similar, analysis would be required for weaker assumptions on *f*. Similar considerations apply to the assumptions on $\phi$.

#### Theorem 2.4

Consider the numerical method (10) and assume that Assumptions 1 and 3 are satisfied. Then, for $\phi \in C^{\infty }$ function with all derivatives bounded uniformly on $\mathbb {R}^n$, we have that

$$\begin{aligned} |\phi (u(T))-\mathbb {E}^h\bigl (\phi (U_{k})\bigr ) | \le K h^{\min \{2p,q\}}, \quad kh=T, \end{aligned}$$

and

$$\begin{aligned} |\mathbb {E}\bigl (\phi (\tilde{u}(T))\bigr )-\mathbb {E}^h\bigl (\phi (U_{k})\bigr ) | \le K h^{2p+1}, \quad kh=T, \end{aligned}$$

where *u* and $\tilde{u}$ solve (1) and (17), respectively.

#### Example 2.5

Consider the probabilistic integrator derived from the Euler method in dimension $n=1$. We thus have $q=1$, and we hence set $p=1$. The results in Hairer et al. (2006) allow us to calculate $f^h$ with $l=1$. The preceding theory then leads to strong order of convergence 1, measured relative to the true ODE (1), and weak order 3 relative to the SDE

$$\begin{aligned} \mathrm{d}\hat{u}&=\bigg (f(\hat{u})-\frac{h}{2}f'(\hat{u})f(\hat{u})+\frac{h^{2}}{12}\big (f''(\hat{u})f^{2}(\hat{u})\\&\qquad \, +\,4 (f'(\hat{u}))^{2}f(\hat{u})\big )\bigg )\mathrm{d}t+\sqrt{C}h\mathrm{d}W. \end{aligned}$$

These results allow us to constrain the behaviour of the randomised method using limited information about the covariance structure, $C^h$. The randomised solution converges weakly, at a high rate, to a solution that only depends on *Q*. Hence, we conclude that the practical behaviour of the solution is only dependent upon *Q*, and otherwise, $C^h$ may be any convenient kernel. With these results now available, the following section provides an empirical study of our probabilistic integrators.

## Statistical inference and numerics

This section explores applications of the randomised ODE solvers developed in Sect. 2 to forward and inverse problems. Throughout this section, we use the FitzHugh–Nagumo model to illustrate ideas (Ramsay et al. 2007). This is a two-state non-linear oscillator, with states (*V*, *R*) and parameters (*a*, *b*, *c*), governed by the equations

29 $$\begin{aligned} \frac{\mathrm{d}V}{\mathrm{d}t} = c \left( V - \frac{V^3}{3} +R \right) ,\;\;\; \frac{\mathrm{d}R}{\mathrm{d}t} = -\frac{1}{c}\left( V-a+bR \right) . \end{aligned}$$

This particular example does not satisfy the stringent Assumptions 2 and 3 and the numerical results shown demonstrate that, as indicated in Remarks 2.1 and 2.3, our theory will extend to weaker assumptions on *f*, something we will address in future work.

### Calibrating forward uncertainty propagation

**Fig. 3:**
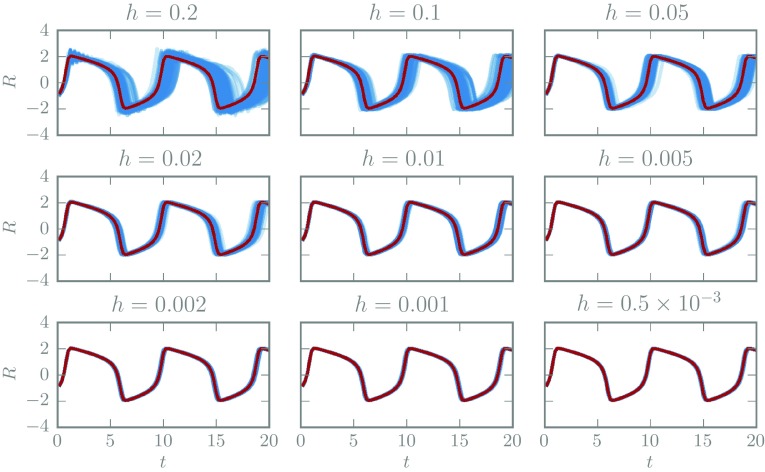
The true trajectory of the *V* species of the FitzHugh–Nagumo model (*red*) and one hundred realisations from a probabilistic Euler ODE solver with various step-sizes and noise scale $\sigma =.1$ (*blue*)

Consider Eq. (29) with fixed initial conditions $V(0)=-1, R(0)=1$, and parameter values (.2, .2, 3). Figure 3 shows draws of the *V* species trajectories from the measure associated with the probabilistic Euler solver with $p=q=1$, for various values of the step-size and fixed $\sigma =0.1$. The random draws exhibit non-Gaussian structure at large step-size and clearly contract towards the true solution.

**Fig. 4:**
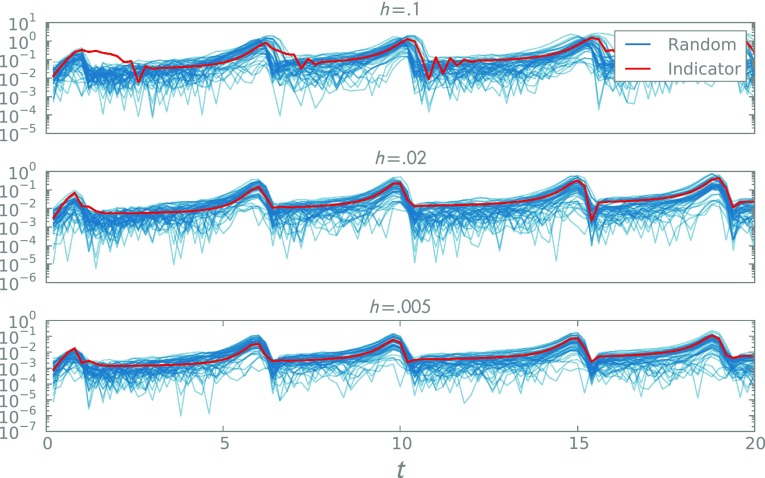
A comparison of the error indicator for the *V* species of the FitzHugh–Nagumo model (*blue*) and the observed variation in the calibrated probabilistic solver. The red curves depict 50 samples of the magnitude of the difference between a standard Euler solver for several step-sizes and the equivalent randomised variant, using $\sigma ^*$, maximising $\pi (\sigma )$

Although the rate of contraction is governed by the underlying deterministic method, the scale parameter, $\sigma$, completely controls the apparent uncertainty in the solver.^6^ This tuning problem exists in general, since $\sigma$ is problem dependent and cannot obviously be computed analytically.

Therefore, we propose to calibrate $\sigma$ to replicate the amount of error suggested by classical error indicators. In the following discussion, we often explicitly denote the dependence on *h* and $\sigma$ with superscripts, hence the probabilistic solver is $U^{h, \sigma }$ and the corresponding deterministic solver is $U^{h, 0}$. Define the deterministic error as $e(t) = u(t) - U^{h, 0}(t)$. Then we assume there is some computable error indicator $E(t) \approx e(t)$, defining $E_k = E(t_k)$. The simplest error indicators might compare differing step-sizes, $E(t) = U^{h, 0}(t) - U^{2h, 0}(t)$, or differing order methods, as in a Runge–Kutta 4–5 scheme.

We proceed by constructing a probability distribution $\pi (\sigma )$ that is maximised when the desired matching occurs. We estimate this scale matching by comparing: (i) a Gaussian approximation of our random solver at each step *k*, $\tilde{\mu }_k^{h,\sigma } = \mathcal {N}(\mathbb {E} (U^{h, \sigma }_k), \mathbb {V}(U^{h, \sigma }_k ));$ and (ii) the natural Gaussian measure from the deterministic solver, $U^{h, 0}_k$, and the available error indicator, $E_k$, $\nu _k^\sigma = \mathcal {N}(U^{h, 0}_k, (E_k)^2).$ We construct $\pi (\sigma )$ by penalising the distance between these two normal distributions at every step: $\pi (\sigma ) \propto \prod _k \exp \left( -d(\tilde{\mu }_k^{h,\sigma }, \nu _k^\sigma ) \right)$. We find that the Bhattacharyya distance (closely related to the Hellinger metric) works well (Kailath 1967), since it diverges quickly if either the mean or variance differs. The density can be easily estimated using Monte Carlo. If the ODE state is a vector, we take the product of the univariate Bhattacharyya distances. Note that this calibration depends on the initial conditions and any parameters of the ODE.

Returning to the FitzHugh–Nagumo model, sampling from $\pi (\sigma )$ yields strongly peaked, uni-modal posteriors, hence we proceed using $\sigma ^*= \hbox {arg max}\pi (\sigma )$. We examine the quality of the scale matching by plotting the magnitudes of the random variation against the error indicator in Fig. 4, observing good agreement of the marginal variances. Note that our measure still reveals non-Gaussian structure and correlations in time not revealed by the deterministic analysis. As described, this procedure requires fixed inputs to the ODE, but it is straightforward to marginalise out a prior distribution over input parameters.

### Bayesian posterior inference problems

Given the calibrated probabilistic ODE solvers described above, let us consider how to incorporate them into inference problems.

Assume we are interested in inferring parameters of the ODE given noisy observations of the state. Specifically, we wish to infer parameters $$\theta \in \mathbb {R}^d$$ for the differential equation $$\dot{u} = f(u, \theta )$$, with fixed initial conditions $$u(t=0) = u_0 $$ (a straightforward modification may include inference on initial conditions). Assume we are provided with data $$d \in \mathbb {R}^m$$, $$d_j = u(\tau _j) + \eta _j$$ at some collection of times $$\tau _j$$, corrupted by i.i.d. noise, $$\eta _j \sim \mathcal {N}(0, \varGamma )$$. If we have prior $$\mathbb {Q}(\theta )$$, the posterior we wish to explore is, $$ \mathbb {P}(\theta \mid d) \propto \mathbb {Q}(\theta ) \mathcal {L}(d,u(\theta )), $$ where density $$\mathcal {L}$$ compactly summarises this likelihood model.

The standard computational strategy is to simply replace the unavailable trajectory *u* with a numerical approximation, inducing approximate posterior $\mathbb {P}^{h, 0}(\theta \mid d) \propto \mathbb {Q}(\theta ) \mathcal {L}(d,U^{h, 0}(\theta )).$ Informally, this approximation will be accurate when the error in the numerical solver is small compared to $\varGamma$ and often converges formally to $\mathbb {P}(\theta \mid d)$ as $h \rightarrow 0$ (Dashti and Stuart 2016). However, highly correlated errors at finite *h* can have substantial impact.

In this work, we are concerned about the undue optimism in the predicted variance, that is, when the posterior concentrates around an arbitrary parameter value even though the deterministic solver is inaccurate and is merely able to reproduce the data by coincidence. The conventional concern is that any error in the solver will be transferred into posterior bias. Practitioners commonly alleviate both concerns by tuning the solver to be nearly perfect, however, we note that this may be computationally prohibitive in many contemporary statistical applications.

We can construct a different posterior that includes the uncertainty in the solver by taking an expectation over random solutions to the ODE

30 $$\begin{aligned} \mathbb {P}^{h, \sigma }(\theta \mid d) \propto \mathbb {Q}(\theta ) \int \mathcal {L}(d,U^{h, \sigma }(\theta , \xi )) d\xi , \end{aligned}$$

where $U^{h, \sigma }(\theta , \xi )$ is a draw from the randomised solver given parameters $\theta$ and random draw $\xi$. Intuitively, this construction favours parameters that exhibit agreement with the entire family of uncertain trajectories. The typical effect of this expectation is to increase the posterior uncertainty on $\theta$, preventing the inappropriate posterior collapse we are concerned about. Indeed, if the integrator cannot resolve the underlying dynamics, $h^{p+1/2}\sigma$ will be large. Then $U^{h, \sigma }(\theta , \xi )$ is independent of $\theta$, hence the prior is recovered, $\mathbb {P}^{h, \sigma }(\theta \mid d) \approx \mathbb {Q}(\theta )$.

Notice that as $h \rightarrow 0$, both the measures $\mathbb {P}^{h, 0}$ and $\mathbb {P}^{h, \sigma }$ typically collapse to the analytic posterior, $\mathbb {P}$, hence both methods are correct. We do not expect the bias of $\mathbb {P}^{h, \sigma }$ to be improved, since all of the averaged trajectories are of the same quality as the deterministic solver in $\mathbb {P}^{h, 0}$. We now construct an analytic inference problem demonstrating these behaviours.

#### Example 3.1

Consider inferring the initial condition, $u_0\in \mathbb {R}$, of the scalar linear differential equation, $\dot{u} = \lambda u,$ with $\lambda >0.$ We apply a numerical method to produce the approximation $U_k \approx u(kh)$. We observe the state at some times $t=kh$, with additive noise $\eta _k \sim \mathcal {N}(0, \gamma ^2)$: $d_k = U_k + \eta _k$. If we use a deterministic Euler solver, the model predicts $U_{k} = (1+h\lambda )^k u_0.$ These model predictions coincide with the slightly perturbed problem

$$\begin{aligned} \frac{\mathrm{d}u}{\mathrm{d}t} = h^{-1}\log (1+\lambda h)u, \end{aligned}$$

hence error increases with time. However, the assumed observational model does not allow for this, as the observation variance is $\gamma ^2$ at all times.

In contrast, our proposed probabilistic Euler solver predicts

$$\begin{aligned} U_{k} = (1+h\lambda )^k u_0 + \sigma h^{3/2} \sum _{j=0}^{k-1} \xi _j (1+\lambda h)^{k-j-1}, \end{aligned}$$

where we have made the natural choice $p=q$, where $\sigma$ is the problem-dependent scaling of the noise and the $\xi _k$ are i.i.d. $\mathcal {N}(0,1).$ For a single observation, $\eta _k$ and every $\xi _k$ are independent, so we may rearrange the equation to consider the perturbation as part of the observation operator. Hence, a single observation at *k* has effective variance

$$\begin{aligned} \gamma _h^2:= & {} \gamma ^2 + \sigma ^2 h^{3} \sum _{j=0}^{k-1} (1+\lambda h)^{2(k-j-1)}\\= & {} \gamma ^2+\sigma ^2 h^{3}\frac{(1+\lambda h)^{2k}-1}{(1+\lambda h)^2-1}. \end{aligned}$$

Thus, late-time observations are modelled as being increasingly inaccurate.

Consider inferring $u_0$, given a single observation $d_k$ at time *k*. If a Gaussian prior $\mathcal {N}(m_0,\zeta _0^2)$ is specified for $u_0$, then the posterior is $\mathcal {N}(m,\zeta ^2)$, where

$$\begin{aligned}&\zeta ^{-2}=\frac{(1+h\lambda )^{2k}}{\gamma _h^2}+\zeta _0^{-2}, \qquad \\&\zeta ^{-2}m=\frac{(1+h\lambda )^k d_k}{\gamma _h^2}+\zeta _0^{-2}m_0. \end{aligned}$$

The observation precision is scaled by $(1+h\lambda )^{2k}$ because late-time data contain increasing information. Assume that the data are $d_k=e^{\lambda kh}u_0^{\dagger }+\gamma \eta ^{\dagger }$, for some given true initial condition $u_0^{\dagger }$ and noise realisation $\eta ^{\dagger }.$ Consider now the asymptotic regime, where *h* is fixed and $k \rightarrow \infty$. For the standard Euler method, where $\gamma _h=\gamma$, we see that $\zeta ^2 \rightarrow 0$, whilst $m \asymp \bigl ((1+h\lambda )^{-1}e^{h\lambda }\bigr )^{k} u_0^\dagger$. Thus the inference scheme becomes increasingly certain of the wrong answer: the variance tends to zero and the mean tends to infinity.

In contrast, with a randomised integrator, the fixed *h*, large *k* asymptotics are

$$\begin{aligned}&\zeta ^2 \asymp \frac{1}{\zeta _0^{-2}+\lambda (2+\lambda h)\sigma ^{-2}h^{-2}}, \\&m \asymp \frac{\left( (1+h\lambda )^{-1}e^{h\lambda }\right) ^{k} u_0^\dagger }{1+\zeta _0^{-2}\sigma ^2h^2\lambda ^{-1}(2+\lambda h)^{-1}}. \end{aligned}$$

Thus, the mean blows up at a modified rate, but the variance remains positive.

We take an empirical Bayes approach to choosing $\sigma$, that is, using a constant, fixed value $\sigma ^*= \hbox {arg max}\pi (\sigma )$, chosen before the data are observed. Joint inference of the parameters and the noise scale suffer from well-known MCMC mixing issues in Bayesian hierarchic models. To handle the unknown parameter $\theta$, we can marginalise it out using the prior distribution, or in simple problems, it may be reasonable to choose a fixed representative value.

We now return to the FitzHugh–Nagumo model; given fixed initial conditions, we attempt to recover parameters $\theta = (a,b,c)$ from observations of both species at times $\tau = 1,2,\ldots ,40$. The priors are log-normal, centred on the true value with unit variance, and with observational noise $\varGamma = 0.001$. The data are generated from a high-quality solution, and we perform inference using Euler integrators with various step-sizes, $h \in \{0.005, 0.01, 0.02, 0.05, 0.1\}$, spanning a range of accurate and inaccurate integrators.

**Fig. 5:**
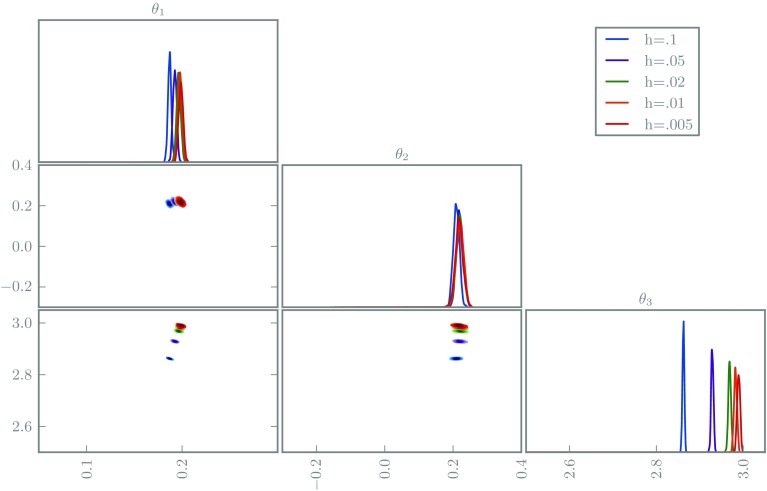
The posterior marginals of the FitzHugh–Nagumo inference problem using deterministic integrators with various step-sizes

We first perform the inferences with naive use of deterministic Euler integrators. We simulate from each posterior using delayed rejection MCMC (Haario et al. 2006), shown in Fig. 5. Observe the undesirable concentration of every posterior, even those with poor solvers; the posteriors are almost mutually singular, hence clearly the posterior widths are meaningless.

**Fig. 6:**
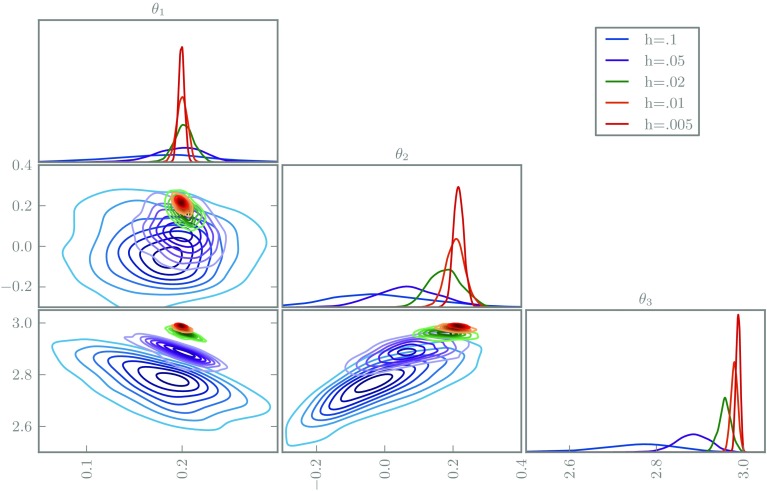
The posterior marginals of the FitzHugh–Nagumo inference problem using probabilistic integrators with various step-sizes

Secondly, we repeat the experiment using our probabilistic Euler integrators, with results shown in Fig. 6. We use a noisy pseudomarginal MCMC method, whose fast mixing is helpful for these initial experiments (Medina-Aguayo et al. 2015). These posteriors are significantly improved, exhibiting greater mutual agreement and obvious increasing concentration with improving solver quality. The posteriors are not perfectly nested, possible evidence that our choice of scale parameter is imperfect, or that the assumption of locally Gaussian error deteriorates for large step-sizes. Note that the bias of $\theta _3$ is essentially unchanged with the randomised integrator, but the posterior for $\theta _2$ broadens and is correlated to $\theta _3$, hence introduces a bias in the posterior mode; without randomisation, only the inappropriate certainty about $\theta _3$ allowed the marginal for $\theta _2$ to exhibit little bias.

## Probabilistic solvers for partial differential equations

We now turn to present a framework for probabilistic solutions to partial differential equations, working within the finite element setting. Our discussion closely resembles the ODE case, except that now we randomly perturb the finite element basis functions.

### Probabilistic finite element method for variational problems

Let $\mathcal {V}$ be a Hilbert space of real-valued functions defined on a bounded polygonal domain $D \subset \mathbb {R}^d$. Consider a weak formulation of a linear PDE specified via a symmetric bilinear form $a:\mathcal {V}\times \mathcal {V}\longrightarrow \mathbb {R}$, and a linear form $r:\mathcal {V}\longrightarrow \mathbb {R}$ to give the problem of finding $u\in \mathcal {V}: \, a(u,v)=r(v), \quad \forall v\in \mathcal {V}.$ This problem can be approximated by specifying a finite-dimensional subspace $\mathcal {V}^h \subset \mathcal {V}$ and seeking a solution in $\mathcal {V}^h$ instead. This leads to a finite-dimensional problem to be solved for the approximation *U*:

31 $$\begin{aligned} U\in \mathcal {V}^h: a(U,v)=r(v), \quad \forall v\in \mathcal {V}^h. \end{aligned}$$

This is known as the Galerkin method.

We will work in the setting of finite element methods, assuming that $\mathcal {V}^h=\mathrm {span}\{\phi _j\}_{j=1}^J$, where $\phi _j$ is locally supported on a grid of points $\{x_j\}_{j=1}^J.$ The parameter *h* is introduced to measure the diameter of the finite elements. We will also assume that

32 $$\begin{aligned} \phi _j(x_k)=\delta _{jk}. \end{aligned}$$

Any element $U \in \mathcal {V}^h$ can then be written as

33 $$\begin{aligned} U(x)=\sum _{j=1}^{J} U_j \phi _j(x) \end{aligned}$$

from which it follows that $U(x_k)=U_k.$ The Galerkin method then gives $A\mathsf {U}=\mathsf {r}$, for $\mathsf {U}=(U_1,\ldots , U_J)^T$, $A_{jk}=a(\phi _j,\phi _k)$, and $\mathsf {r}_k=r(\phi _k).$

In order to account for uncertainty introduced by the numerical method, we will assume that each basis function $\phi _j$ can be split into the sum of a systematic part $\phi ^{\mathsf {s}}_j$ and random part $\phi ^{\mathsf {r}}_j$, where both $\phi _j$ and $\phi ^{\mathsf {s}}_j$ satisfy the nodal property (32), hence $\phi ^{\mathsf {r}}_j(x_k) = 0$. Furthermore, we assume that each $\phi ^{\mathsf {r}}_j$ shares the same compact support as the corresponding $\phi ^{\mathsf {s}}_j$, preserving the sparsity structure of the underlying deterministic method.

### Strong convergence result

As in the ODE case, we begin our convergence analysis with assumptions constraining the random perturbations and the underlying deterministic approximation. The bilinear form $a(\cdot ,\cdot )$ is assumed to induce an inner product, and then norm via $\Vert \cdot \Vert _a^2=a(\cdot ,\cdot );$ furthermore, we assume that this norm is equivalent to the norm on $\mathcal {V}$. Throughout, $\mathbb {E}^h$ denotes expectation with respect to the random basis functions.

#### Assumption 4

The collection of random basis functions $\{\phi ^{\mathsf {r}}_j\}_{j=1}^J$ are independent, zero-mean, Gaussian random fields, each of which satisfies $\phi ^{\mathsf {r}}_j(x_k)=0$ and shares the same support as the corresponding systematic basis function $\phi ^{\mathsf {s}}_j.$ For all *j*, the number of basis functions with index *k* which share the support of the basis functions with index *j* is bounded independently of *J*, the total number of basis functions. Furthermore, the basis functions are scaled so that $\sum _{j=1}^J \mathbb {E}^h\Vert \phi ^{\mathsf {r}}_j\Vert _a^2 \le Ch^{2p}.$

#### Assumption 5

The true solution *u* of problem (4.1) is in $L^{\infty }(D).$ Furthermore, the standard deterministic interpolant of the true solution, defined by $v^{\mathsf {s}}:=\sum _{j=1}^J u(x_j)\phi ^{\mathsf {s}}_j,$ satisfies $\Vert u-v^{\mathsf {s}}\Vert _a \le Ch^q.$

#### Theorem 4.1

Under Assumptions 4 and 5 it follows that the approximation *U*, given by (31), satisfies

$$\begin{aligned} \mathbb {E}^h\Vert u-U\Vert _a^2 \le Ch^{2\min \{p,q\}}. \end{aligned}$$

As for ODEs, the solver accuracy is limited by either the amount of noise injected or the convergence rate of the underlying deterministic method, making $p=q$ the natural choice.

### Poisson solver in two dimensions

Consider a Poisson equation with Dirichlet boundary conditions in dimension $d=2$, namely

$$\begin{aligned}&-\triangle u=f, \quad x \in D,\\&u=0, \quad x \in \partial D. \end{aligned}$$

We set $\mathcal {V}=H^1_0(D)$ and *H* to be the space $L^2(D)$ with inner product $\langle \cdot , \cdot \rangle$ and resulting norm $| \cdot |^2=\langle \cdot , \cdot \rangle .$ The weak formulation of the problem has the form (4.1) with

$$\begin{aligned} a(u,v)=\int _{D} \nabla u(x) \nabla v(x) \mathrm{d}x, \quad r(v)=\langle f,v \rangle . \end{aligned}$$

Now consider piecewise linear finite elements satisfying the assumptions of Sect. 4.2 in Johnson (2012) and take these to comprise the set $\{\phi ^{\mathsf {s}}_j\}_{j=1}^J.$ Then *h* measures the width of the triangulation of the finite element mesh. Assuming that $f \in H$ it follows that $u \in H^2(D)$ and that

34 $$\begin{aligned} \Vert u-v^{\mathsf {s}}\Vert _a \le Ch\Vert u\Vert _{H^2}. \end{aligned}$$

Thus $q=1$. We choose random basis members $\{\phi ^{\mathsf {r}}_j\}_{j=1}^J$ so that Assumption 4 hold with $p=1$. Theorem 4.1 then shows that, for $e=u-U$, $\mathbb {E}^h\Vert e\Vert _a^2 \le Ch^{2}.$ We note that that in the deterministic case, we expect an improved rate of convergence in the function space *H*. Such a result can be shown to hold in our setting, following the usual arguments for the Aubin–Nitsche trick Johnson (2012), which is available in the supplementary materials.

## PDE inference and numerics

We now perform numerical experiments using probabilistic solvers for elliptic PDEs. Specifically, we perform inference in a 1D elliptic PDE, $\nabla \cdot (\kappa (x) \nabla u(x)) = 4x$ for $x \in [0,1]$, given boundary conditions $u(0) = 0, u(1) = 2$. We represent $\log \kappa$ as piecewise constant over ten equal-sized intervals; the first, on $x \in [0,.1)$ is fixed to be one to avoid non-identifiability issues, and the other nine are given a prior $\theta _i = \log \kappa _i \sim \mathcal {N}(0,1)$. Observations of the field *u* are provided at $x=(0.1, 0.2, \ldots 0.9)$, with i.i.d. Gaussian error, $\mathcal {N}(0, 10^{-5})$; the simulated observations were generated using a fine grid and quadratic finite elements, then perturbed with error from this distribution.

Again we investigate the posterior produced at various grid sizes, using both deterministic and randomised solvers. The randomised basis functions are draws from a Brownian bridge conditioned to be zero at the nodal points, implemented in practice with a truncated Karhunen–Loève expansion. The covariance operator may be viewed as a fractional Laplacian, as discussed in Lindgren et al. (2011). The scaling $\sigma$ is again determined by maximising the distribution described in Sect. 3.1, where the error indicator compares linear to quadratic basis functions, and we marginalise out the prior over the $\kappa _i$ values.

**Fig. 7:**
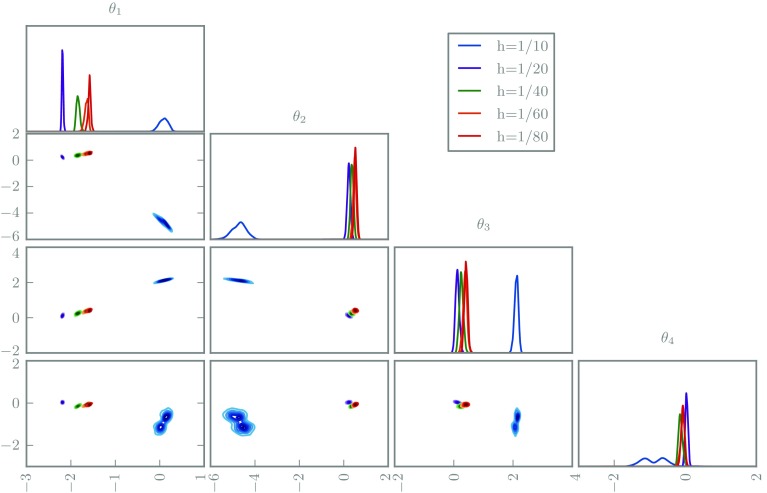
The marginal posterior distributions for the first four coefficients in 1D elliptic inverse problem using a classic deterministic solver with various grid sizes

**Fig. 8:**
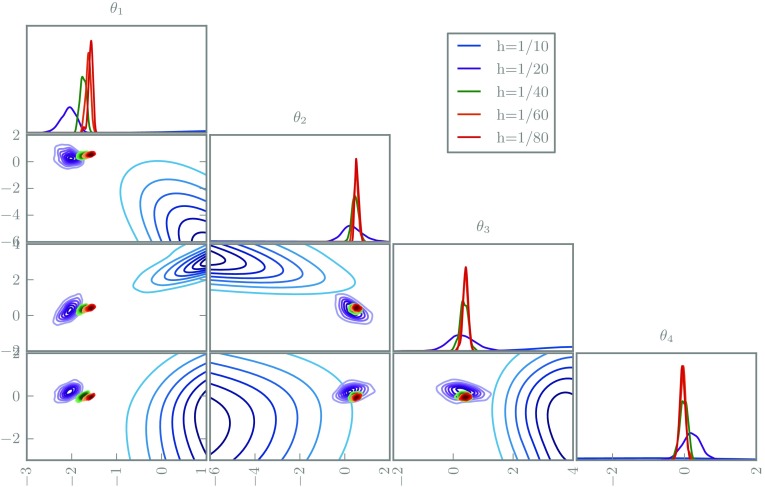
The marginal posterior distributions for the first four coefficients in 1D elliptic inverse problem using a randomised solver with various grid sizes

The posteriors are depicted in Figs. 7 and 8. As in the ODE examples, the deterministic solvers lead to incompatible posteriors for varying grid sizes. In contrast, the randomised solvers suggest increasing confidence as the grid is refined, as desired. The coarsest grid size uses an obviously inadequate ten elements, but this is only apparent in the randomised posterior.

## Conclusions

We have presented a computational methodology, backed by rigorous analysis, which enables quantification of the uncertainty arising from the finite-dimensional approximation of solutions of differential equations. These methods play a natural role in statistical inference problems as they allow for the uncertainty from discretisation to be incorporated alongside other sources of uncertainty such as observational noise. We provide theoretical analyses of the probabilistic integrators which form the backbone of our methodology. Furthermore we demonstrate empirically that they induce more coherent inference in a number of illustrative examples. There are a variety of areas in the sciences and engineering which have the potential to draw on the methodology introduced including climatology, computational chemistry, and systems biology.

Our key strategy is to make assumptions about the *local* behaviour of solver error, which we have assumed to be Gaussian, and to draw samples from the *global* distribution of uncertainty over solutions that results. Section 2.4 describes a universality result, simplifying task of choosing covariance kernels in practice, within the family of Gaussian processes. However, assumptions of Gaussian error, even locally, may not be appropriate in some cases, or may neglect important domain knowledge. Our framework can be extended in future work to consider alternate priors on the error, for example, multiplicative or non-negative errors.

Our study highlights difficult decisions practitioners face, regarding how to expend computational resources. While standard techniques perform well when the solver is highly converged, our results show standard techniques can be disastrously wrong when the solver is not converged. As the measure of convergence is not a standard numerical analysis one, but a statistical one, we have argued that it can be surprisingly difficult to determine in advance which regime a particular problem resides in. Therefore, our practical recommendation is that the lower cost of the standard approach makes it preferable when it is certain that the numerical method is strongly converged with respect to the statistical measure of interest. Otherwise, the randomised method we propose provides a robust and consistent approach to address the error introduced into the statistical task by numerical solver error. In difficult problem domains, such as numerical weather prediction, the focus has typically been on reducing the numerical error in each solver run; techniques such as these may allow a difference balance between numerical and statistical computing effort in the future.

The prevailing approach to model error described in Kennedy and O’Hagan (2001) is based on a non-intrusive methodology where the effect of model discrepancy is allowed for in observation space. Our intrusive randomisation of deterministic methods for differential equations can be viewed as a highly specialised discrepancy model, designed using our intimate knowledge of the structure and properties of numerical methods. In this vein, we intend to extend this work to other types of model error, where modifying the internal structure of the models can produce computationally and analytically tractable measures of uncertainty which perform better than non-intrusive methods. Our future work will continue to study the computational challenges and opportunities presented by these techniques.

### Electronic supplementary material

Below is the link to the electronic supplementary material.

Supplementary material 1 (tex 5 KB)

## A Numerical analysis details and proofs

### Proof

(Theorem 2.2) We first derive the convergence result on the grid, and then in continuous time. From (10) we have

35 $$\begin{aligned} U_{k+1}=\varPsi _{h}(U_{k})+\xi _k(h) \end{aligned}$$

whilst we know that

36 $$\begin{aligned} u_{k+1}=\varPhi _h(u_{k}). \end{aligned}$$

Define the truncation error $\epsilon _k=\varPsi _{h}(U_{k})-\varPhi _h(U_k)$ and note that

37 $$\begin{aligned} U_{k+1}=\varPhi _h(U_k)+\epsilon _k+\xi _k(h). \end{aligned}$$

Subtracting Eq. (37) from (36) and defining $e_k=u_k-U_k$, we get

$$\begin{aligned} e_{k+1} = \varPhi _h(u_{k})-\varPhi _h(u_{k}-e_{k})-\epsilon _k-\xi _k(h). \end{aligned}$$

Taking the Euclidean norm and expectations give, using Assumption 1 and the independence of the $\xi _k$,

$$\begin{aligned} \mathbb {E}^h|e_{k+1}|^2 =\mathbb {E}^h\Bigl |\varPhi _h(u_{k})-\varPhi _h(u_{k}-e_{k})-\epsilon _k\Bigr |^2+{\mathcal {O}}(h^{2p+1}), \end{aligned}$$

where the constant in the ${\mathcal {O}}(h^{2p+1})$ term is uniform in $k: 0 \le kh \le T.$ Assumption 2 implies that $\epsilon _k={\mathcal {O}}(h^{q+1}),$ again uniformly in $k: 0 \le kh \le T.$ Noting that $\varPhi _h$ is globally Lipschitz with constant bounded by $1+Lh$ under Assumption 2, we then obtain

$$\begin{aligned} \mathbb {E}^h|e_{k+1}|^2\le & {} (1+Lh)^2 \mathbb {E}^h|e_k|^2\\&+\, \mathbb {E}^h\Bigl |\bigl \langle h^{\frac{1}{2}}\bigl (\varPhi _h(u_{k}) -\varPhi _h(u_{k}-e_{k})\bigr ),h^{-\frac{1}{2}}\epsilon _k \bigr \rangle \Bigr |\\&+\,{\mathcal {O}}(h^{2q+2})+{\mathcal {O}}(h^{2p+1}). \end{aligned}$$

Using Cauchy–Schwarz on the inner product, and the fact that $\varPhi _h$ is Lipschitz with constant bounded independently of *h*, we get

$$\begin{aligned} \mathbb {E}^h|e_{k+1}|^2 \le \bigl (1+{\mathcal {O}}(h)\bigr )\mathbb {E}^h|e_k|^2+ {\mathcal {O}}(h^{2q+1})+{\mathcal {O}}(h^{2p+1}). \end{aligned}$$

Application of the Gronwall inequality gives the desired result.

Now we turn to continuous time. We note that, for $s \in [t_k,t_{k+1})$,

$$\begin{aligned} U(s)&=\varPsi _{s-t_k}(U_k)+\xi _k(s-t_k),\\ u(s)&=\varPhi _{s-t_k}(u_k). \end{aligned}$$

Let $\mathcal {F}_t$ denote the $\sigma$-algebra of events generated by the $\{\xi _k\}$ up to time *t*. Subtracting we obtain, using Assumptions 1 and 2 and the fact that $\varPhi _{s-t_k}$ has Lipschitz constant of the form $1+{\mathcal {O}}(h)$,

$$\begin{aligned}&\mathbb {E}^h\bigl (|U(s)-u(s)|\big | \mathcal {F}_{t_k}\bigr )\\&\qquad \le |\varPhi _{s-t_k}(U_k)-\varPhi _{s-t_k}(u_k)| \\&\qquad \quad + |\varPsi _{s-t_k}(U_k)-\varPhi _{s-t_k}(U_k)|\\&\qquad \quad +\mathbb {E}^h\bigl (|\xi _k(s-t_k)|\big |\mathcal {F}_{t_k}\bigr )\\&\qquad \le (1+Lh)|e_k|+{\mathcal {O}}(h^{q+1})+\mathbb {E}^h|\xi _k(s-t_k)|\\&\qquad \le (1+Lh)|e_k|+{\mathcal {O}}(h^{q+1})\\&\qquad \quad + \bigl (\mathbb {E}^h|\xi _k(s-t_k)|^2\bigr )^{\frac{1}{2}}\\&\qquad \le (1+Lh)|e_k|+{\mathcal {O}}(h^{q+1}) +{\mathcal {O}}\left( h^{p+\frac{1}{2}}\right) . \end{aligned}$$

Now taking expectations we obtain

$$\begin{aligned} \mathbb {E}^h|U(s)-u(s)|\le & {} (1+Lh)\bigl (\mathbb {E}^h|e_k|^2\bigr )^{\frac{1}{2}}+{\mathcal {O}}(h^{q+1})\\&+\,{\mathcal {O}}\left( h^{p+\frac{1}{2}}\right) . \end{aligned}$$

Using the on-grid error bound gives the desired result, after noting that the constants appearing are uniform in $0 \le kh \le T.$ $\square$

### Proof

(Theorem 2.4) We prove the second bound first. Let $w_k=\mathbb {E}\bigl (\phi (\tilde{u}(t_k))| \tilde{u}(0)=u\bigr )$ and $W_k=\mathbb {E}^h\bigl (\phi (U_{k})|U_0=u).$ Then let $\delta _k=\sup _{u \in \mathbb {R}^n}|W_k-w_k|.$ It follows from the Markov property that

$$\begin{aligned} W_{k+1}-w_{k+1}&=e^{h\mathcal {L}^h}W_k-e^{h\widetilde{\mathcal {L}}^h}w_k\\&=e^{h\mathcal {L}^h}W_k-e^{h\mathcal {L}^h}w_k\\&\quad +\bigl (e^{h\mathcal {L}^h}w_k-e^{h\widetilde{\mathcal {L}}^h}w_k). \end{aligned}$$

Using (28) and Assumption 3 we obtain

$$\begin{aligned} \delta _{k+1} \le (1+Lh)\delta _k+\mathcal {O}(h^{2p+2}). \end{aligned}$$

Iterating and employing the Gronwall inequality gives the second error bound.

Now we turn to the first error bound, comparing with the solution *u* of the original Eq. (1). From (25) and then (21) we see that

$$\begin{aligned} e^{h \mathcal {L}^h}\phi (u)-\phi (\varPsi _{h}(u))&=\mathcal {O}(h^{2p+1}),\\ e^{h\mathcal {L}}\phi (u)-e^{h\mathcal {L}^h}\phi (u)&=\mathcal {O}(h^{\min \{2p+1,q+1\}}). \end{aligned}$$

This gives the first weak error estimate, after using the stability estimate on $e^{h\mathcal {L}}$ from Assumption 3. $\square$

**Proof **(Theorem 4.1) Recall the Galerkin orthogonality property which follows from subtracting the approximate variational principle from the true variational principle: it states that, for $e=u-U$,

38 $$\begin{aligned} a(e,v)= 0, \quad \forall v\in \mathcal {V}^h. \end{aligned}$$

From this it follows that

39 $$\begin{aligned} \Vert e\Vert _{a} \le \Vert u-v\Vert _{a}, \quad \forall v \in \mathcal {V}^h. \end{aligned}$$

To see this note that, for any $v \in \mathcal {V}^h$, the orthogonality property (38) gives

40 $$\begin{aligned} a(e,e) = a(e,e+U-v)= a(e,u-v). \end{aligned}$$

Thus, by Cauchy–Schwarz, $\Vert e\Vert _{a}^2 \le \Vert e\Vert _{a}\Vert u-v\Vert _{a}, \quad \forall v \in \mathcal {V}^h$ implying (39). We now set, for $v \in \mathcal {V}$,

$$\begin{aligned} v(x)= & {} \sum _{j=1}^J u(x_j)\phi _j(x)\\= & {} \sum _{j=1}^J u(x_j)\phi ^{\mathsf {s}}_j(x)+\sum _{j=1}^J u(x_j)\phi ^{\mathsf {r}}_j(x)\\=: & {} v^{\mathsf {s}}(x)+v^{\mathsf {r}}(x). \end{aligned}$$

By the mean-zero and independence properties of the random basis functions we deduce that

$$\begin{aligned} \mathbb {E}^h\Vert u-v\Vert _a^2= & {} \mathbb {E}^ha(u-v,u-v)\\= & {} \mathbb {E}^ha(u-v^{\mathsf {s}},u-v^{\mathsf {s}})+\mathbb {E}^ha(v^{\mathsf {r}},v^{\mathsf {r}})\\= & {} \Vert u-v^{\mathsf {s}}\Vert _a^2+\sum _{j=1}^J u(x_j)^2 \mathbb {E}^h\Vert \phi ^{\mathsf {r}}_j\Vert _a^2. \end{aligned}$$

The result follows from Assumptions 4 and 5. $\square$

## Ornstein–Uhlenbeck integrator

An additional example of a randomised integrator is an *integrated Ornstein–Uhlenbeck process*, derived as follows. Define, on the interval $s \in [t_k,t_{k+1})$, the pair of equations

41a $$\begin{aligned} \mathrm{d}U&=V\mathrm{d}t, \quad U(t_k)=U_k, \end{aligned}$$

41b $$\begin{aligned} \mathrm{d}V&=-\varLambda V\mathrm{d}t+\sqrt{2\varSigma } \, \mathrm{d}W, \quad V(t_k)=f(U_k). \end{aligned}$$

Here *W* is a standard Brownian motion and $\varLambda$ and $\varSigma$ are invertible matrices, possibly depending on *h*. The approximating function $g^h(s)$ is thus defined by *V*(*s*), an Ornstein–Uhlenbeck process.

Integrating (41b) we obtain

42 $$\begin{aligned} V(s)=\exp \bigl (-\varLambda (s-t_k)\bigr )f(U_{k})+\chi _k(s-t_k), \end{aligned}$$

where $s \in [t_k,t_{k+1})$ and the $\{\chi _k\}$ form an i.i.d. sequence of Gaussian random functions defined on [0, *h*] with

$$\begin{aligned} \chi _k(s)=\sqrt{2\varSigma }\int _0^{s}\exp \bigl (\varLambda (\tau -s)\bigr ) \, \mathrm{d}W(\tau ). \end{aligned}$$

Note that the *h*-dependence of $C^h$ comes through the time interval on which $\chi _k$ is defined, and through $\varLambda$ and $\varSigma$.

Integrating (41a), using (42), we obtain

43 $$\begin{aligned} U(s)= & {} U_k+\varLambda ^{-1}\left( I\,-\,\exp \bigl (-\varLambda (s\,-\,t_k)\bigr )\right) f(U_{k})\nonumber \\&+\,\xi _k(s-t_k), \end{aligned}$$

where $s \in [t_k,t_{k+1}]$, and, for $t \in [0,h]$,

44 $$\begin{aligned} \xi _k(t)=\int _{0}^{t}\chi _{k}(\tau )\mathrm{d}\tau . \end{aligned}$$

The numerical method (43) may be written in the form (12), and hence (10) at the grid points, with the definition

$$\begin{aligned} \varPsi _{h}(u)=u+\varLambda ^{-1}\Bigl (I-\exp \bigl (-\varLambda h\bigr )\Bigr )f(u). \end{aligned}$$

This integrator is first-order accurate and satisfies Assumption 2 with $p=1$. Choosing to scale $\varSigma$ with *h* so that $q \ge 1$ in Assumption 1 leads to convergence of the numerical method with order 1.

Had we carried out the above analysis in the case $\varLambda =0$ we would have obtained the probabilistic Euler method (14), and hence (13) at grid points, used as our canonical example in the earlier developments.

## Convergence rate of $L_2$ convergence

When considering the Poisson problem in two dimensions, as discussed in Sect. 4, we expect an improved rate of convergence in the function space *H*. We now show that such a result also holds in our random setting.

Note that, under Assumption 4, if we introduce $\gamma _{jk}$ that is 1 when two basis functions have overlapping support, and 0 otherwise, then $\gamma _{jk}$ is symmetric and there is constant *C*, independent of *j* and *J*, such that $\sum _{k=1}^J \gamma _{jk} \le C.$ Now let $\varphi$ solve the equation $a(\varphi ,v)=\langle e,v \rangle , \quad \forall v \in \mathcal {V}.$ Then $\Vert \varphi \Vert _{H^2} \le C|e|.$ We define $\varphi ^{\mathsf {s}}$ and $\varphi ^{\mathsf {r}}$ in analogy with the definitions of $v^{\mathsf {s}}$ and $v^{\mathsf {r}}$. Following the usual arguments for application of the Aubin–Nitsche trick Johnson (2012), we have $|e|^2=a(e,\varphi )=a(e,\varphi -\varphi ^{\mathsf {s}}-\varphi ^{\mathsf {r}}).$ Thus

45 $$\begin{aligned} |e|^2\le & {} \Vert e\Vert _a\Vert \varphi \,-\,\varphi ^{\mathsf {s}}\,-\,\varphi ^{\mathsf {r}}\Vert _a\nonumber \\\le & {} \sqrt{2}\Vert e\Vert _a\Bigl (\Vert \varphi \,-\,\varphi ^{\mathsf {s}}\Vert _a^2\,+\,\Vert \varphi ^{\mathsf {r}}\Vert _a^2\Bigl )^{\frac{1}{2}}. \end{aligned}$$

We note that $\varphi ^{\mathsf {r}}(x)=\sum _{j=1}^{J} \varphi (x_j)\phi ^{\mathsf {r}}_j(x)=\Vert \varphi \Vert _{H^2} \sum _{j=1}^{J} a_j \phi ^{\mathsf {r}}_j(x)$ where, by Sobolev embedding ($d=2$ here), $a_j:=\varphi (x_j)/\Vert \varphi \Vert _{H^2}$ satisfies $\max _{1 \le j \le J}|a_j| \le C.$ Note, however, that the $a_j$ are random and correlated with all of the random basis functions. Using this, together with (34), in (45), we obtain

$$\begin{aligned} |e|^2 \le C\Vert e\Vert _a\Bigl (h^2+\bigl \Vert \sum _{j=1}^{J} a_j \phi ^{\mathsf {r}}_j(x)\bigr \Vert _a^2\Bigr )^{\frac{1}{2}}\Vert \varphi \Vert _{H^2}. \end{aligned}$$

We see that

$$\begin{aligned} |e| \le C\Vert e\Vert _a\Bigl (h^2+ \sum _{j=1}^J \sum _{k=1}^J a_ja_k \,a(\phi ^{\mathsf {r}}_j,\phi ^{\mathsf {r}}_k)\Bigr )^{\frac{1}{2}}. \end{aligned}$$

From this and the symmetry of $\gamma _{jk}$, we obtain

$$\begin{aligned} |e|&\le C\Vert e\Vert _a\Bigl (h^2+\sum _{j=1}^J \sum _{k=1}^J \gamma _{jk} \bigl (\Vert \phi ^{\mathsf {r}}_j\Vert _a^2+\Vert \phi ^{\mathsf {r}}_k\Vert _a^2\bigr )\Bigr )^{\frac{1}{2}}\\&\le C\Vert e\Vert _a\Bigl (h^2+2C\sum _{j=1}^J \Vert \phi ^{\mathsf {r}}_j\Vert _a^2\Bigr )^{\frac{1}{2}}. \end{aligned}$$

Taking expectations, using that $p=q=1$, we find, using Assumption 4, that $\mathbb {E}^h|e| \le Ch \bigl (\mathbb {E}^h\bigl \Vert e\Vert _a^2\bigr )^{\frac{1}{2}} \le Ch^2$ as desired. Thus we recover the extra order of convergence over the rate 1 in the $\Vert \cdot \Vert _a$ norm (although the improved rate is in $L^1(\varOmega ;H)$ whilst the lower rate of convergence is in $L^2(\varOmega ;\mathcal {V}).$).

## Footnotes

1 Supplementary materials and code are available online: http://www2.warwick.ac.uk/pints.

2 To simplify our discussion we assume that the ODE is autonomous, that is, *f*(*u*) is independent of time. Analogous theory can be developed for time-dependent forcing.

3 We use $\chi _k \sim N(0,C^h)$ to denote a zero-mean Gaussian process defined on [0, *h*] with a covariance kernel $\mathrm {cov}(\chi _k(t),\chi _k(s)) \triangleq C^h(t,s)$.

4 An additional example of a probabilistic integrator, based on a Ornstein–Uhlenbeck process, is available in the supplementary materials.

5 Implementing Eq. 10 is trivial, since it simply adds an appropriately scaled Gaussian random number after each classical Runge–Kutta step.

6 Recall that throughout we assume that, within the context of Assumption 1, $Q=\sigma I$. More generally it is possible to calibrate an arbitrary positive semi-definite *Q*.
